# Quality of Death in Fighting Bulls during Bullfights: Neurobiology and Physiological Responses

**DOI:** 10.3390/ani11102820

**Published:** 2021-09-27

**Authors:** Daniel Mota-Rojas, Fabio Napolitano, Ana Strappini, Agustín Orihuela, Julio Martínez-Burnes, Ismael Hernández-Ávalos, Patricia Mora-Medina, Antonio Velarde

**Affiliations:** 1 Neurophysiology, Behavior and Animal Welfare Assessment, DPAA, Xochimilco Campus, Universidad Autónoma Metropolitana, Ciudad de México 04960, Mexico; 2 Scuola di Scienze Agrarie, Forestali, Alimentari ed Ambientali, Università Degli Studi Della Basilicata, 85100 Potenza, Italy; fabio.napolitano@unibas.it; 3 Animal Science Institute, Faculty of Veterinary Sciences, Universidad Austral de Chile, Valdivia 5090000, Chile; anastrappini@gmail.com; 4 Facultad de Ciencias Agropecuarias, Universidad Autónoma del Estado de Morelos, Cuernavaca 62209, Mexico; 5 Animal Health Group, Faculty of Veterinary Medicine, Universidad Autónoma de Tamaulipas, Ciudad Victoria 87000, Mexico; jmburnes@docentes.uat.edu.mx; 6 Facultad de Estudios Superiores Cuautitlán, Universidad Nacional Autónoma de México (UNAM), State of Mexico 54714, Mexico; mvziha@hotmail.com (I.H.-Á.); mormed2001@yahoo.com.mx (P.M.-M.); 7 IRTA, Animal Welfare Program, Veinat Sies S-N, 17121 Monells, Spain; antonio.velarde@irta.cat

**Keywords:** pain, abattoir, sensitisation, stunning, cattle, animal welfare, fighting bulls

## Abstract

**Simple Summary:**

Fighting bulls that participate in bullfighting face energy and metabolic demands due to the high intensity and duration of the exercise performed. Under these conditions, specific corporal mechanisms, such as the acid–base balance, are affected, causing metabolic acidosis. However, fighting bulls also undergo muscular injuries, physiological changes, and high enzyme concentrations that reflect the stress to which they are subjected, and in some bulls, bullfights can trigger electrolytic imbalances that include hypercalcaemia, hypermagnesaemia, and hyperphosphataemia, exacerbated by muscular necrosis and myoglobinuria.

**Abstract:**

During bullfights, bulls undergo physiometabolic responses such as glycolysis, anaerobic reactions, cellular oedema, splenic contraction, and hypovolemic shock. The objective of this review article is to present the current knowledge on the factors that cause stress in fighting bulls during bullfights, including their dying process, by discussing the neurobiology and their physiological responses. The literature shows that biochemical imbalances occur during bullfights, including hypercalcaemia, hypermagnesaemia, hyperphosphataemia, hyperlactataemia, and hyperglycaemia, associated with increased endogenous cortisol and catecholamine levels. Creatine kinase, citrate synthase, and lactate dehydrogenase levels also increase, coupled with decreases in pH, blood bicarbonate levels, excess base, partial oxygen pressure, and oxygen saturation. The intense exercise also causes a marked decrease of glycogen in type I and II muscle fibres that can produce myoglobinuria and muscular necrosis. Other observations suggest the presence of osteochondrosis. The existing information allows us to conclude that during bullfights, bulls face energy and metabolic demands due to the high intensity and duration of the exercise performed, together with muscular injuries, physiological changes, and high enzyme concentrations. In addition, the final stage of the bullfight causes a slow dying process for an animal that is sentient and conscious of its surroundings.

## 1. Introduction

Fighting bulls are considered a specialized breed of cattle that has its origins in the species *Bos taurus*, which includes all breeds of bovines involved in various zootechnical practices [1]. As with all domestic bovines, certain criteria exist in the selection of fighting bulls. Since, in this case, the objective is to breed animals that will perform well during bullfights, behavioural characteristics in calves and young bulls that manifest ferocity, aggressiveness, and mobility are among those considered necessary for this spectacle. The selection process of heifers and young bulls involves a testing phase (*tienta* in Spanish), while for bulls, it occurs after an outstanding performance where the bull’s life is spared once the bullfight is over. One goal of these practices is to identify bulls that will fight when provoked by a person using some kind of lure [2]. 

During bullfights, the physiometabolic responses correspond mainly to the presence of different types of stressors, such as physical (tissue injury, pathologies, pain [3]), environmental (extreme weather, microclimate, nutrition, handling [4], transportation [5], noise [6]), and psychosocial factors (social isolation, overcrowding, pain, fear or distress [7]). For fighting bulls, similar to any other mammal, the response degree and the consequence in their homeostasis depends on the stressor type, the duration of the stimulus, and the previous experiences of the animal [8,9]. However, in general, when an external stimulus is perceived as potentially harmful, the central nervous system, through the activation of the sympathoadrenal and hypothalamic–pituitary–adrenal axes (Broom, 2019) and the limbic system [7], triggers a cascade of physiological (i.e., tachycardia, tachypnea, hyperthermia), metabolic (hyperglycemia) [9], endocrine (i.e., stress hormones—catecholamines or glucocorticoids), and behavioral responses. In the short term, these changes serve as an adaptive defense mechanism. However, when an animal cannot maintain its homeostasis due to the process’s chronicity or magnitude, the organism and its health deteriorate along with its welfare [8]. Understanding stress responses in livestock can help refine management procedures and promote the selection of stress-tolerant animals.

The bullfight is divided in three stages called *tercios*: *tercio de varas*, *tercio de banderillas*, and *tercio de muleta*. In the first stage, a lance is stabbed into the bull’s hump, limiting its mobility [10]. The injury inflicted by the lance destroys blood vessels and haemorrhages that can decrease blood volume by 8–18% through perforations of the trapezoid and rhomboid muscles, and the funicular portion of the occipital ligament. In some cases, this injury also affects the accessory nerve and brachial plexus from spinal segments C5, C6, C7, C8, and T1, which control the movement of the thoracic limbs [11,12]. The lance can inflict wounds as deep as 30 cm. If not applied properly, it can compromise the animal’s locomotion, as Barona et al. [10] determined in their analysis of the site, depth, and severity of the lesions produced by this instrument after examining 277 fighting bulls from 43 events. They suggest that those injuries are located, in order of importance, in bull’s shoulders and hump. If the lance penetrates the hindquarters, it compromises the bull’s physical integrity by causing pain in the dorso-lumbar region that reduces its force of locomotion.

In the second stage, the matador stabs six flags (*banderillas*) into the bull’s shoulders and/or hump. This action aggravates the muscle damage already inflicted by the lance because every movement the bull makes while charging the matador and his red cape moves the flags inside the wounds. Their sharp points lacerate muscles in different directions, causing additional haemorrhages [10].

In the third stage, the matador uses a sword to kill the bull by causing profuse bleeding in the thoracic cavity, either by piercing the pleura to cause pneumothorax and the consequent respiratory insufficiency, or the lung or right bronchia, allowing blood to leave the lung, enter the bronchia, and reach the trachea, oesophagus, and upper respiratory pathways [13]. In most cases, the sword also severs the spinal cord’s lateral nerve cords responsible for innervating the thoracic cavity, producing paralysis and respiratory insufficiency. The sword ultimately causes asphyxia by severing of the medulla oblongata or its caudal nervous projections [14,15,16,17]. In this case, the sword cuts blood vessels, the lungs, and the bronchia, causing bronchoaspiration [18]. After the sword, the bull is stabbed with a *puntilla* (short knife) to end the fight, which enters near the first and second cervical vertebrae, it will damage the motor nerves, causing the bull to fall with its limbs extended. If the injury is made near the atlantooccipital joint, the bulb is cut the spinal cord and its caudal nerve projections, possibly leading to the same result [11].

The death of bulls during bullfights—whether by asphyxia or exsanguination—occurs while the animal is fully conscious because the brainstem and/or brain cortex remain intact [17,19].

### 1.1. Stressors of Psychological Origin

Regarding fighting bulls, studies have determined that aggressiveness (animal’s capacity to confront the matador vs. attempting to escape) and ferocity (amount of strength it uses to attack with its entire body, and its resistance to pain) have strong genetic [20] and environment [21] bases.

The temperament of the animal could be another factor that affects the quality of its death. However, studies have determined that even bovines with harsh temperaments (*Bos taurus*, including fighting bulls) and other species eventually become habituated to novel environmental conditions and reduce their behavioural reactivity [22].

The typical handling practices used with fighting bulls require minimising or eliminating contact with humans. In part for this reason, no scientific studies have yet documented the peculiarities of this breed of bulls (*Bos taurus brachyceros*) under these circumstances, though similar results have been determined in *Bos indicus* steers [23], horses [24], and pigs [25,26]. The anatomical and physiological difference observed in the fighting bull have been described in the conformation of the cerebral hemispheres, in the brain weight/carcass weight ratio, and in the size of the cerebral amygdala, observing a negative relationship with respect to the aggressiveness of race [27].

In general, acute severe stress from physical and/or psychological injuries in individuals can induce emotions such as fear or anxiety [28]. During bullfights, factors such as novelty, aggression, and noise, among others, can be stressors that could trigger these emotions [29].

When animals are exposed to situations that they cannot control or are unpredictable (such as isolation, acute noise, or confinement) [30], adaptive hypothalamic, sympathetic, immune, and behavioral responses serve to survive [31]. In farm animals, routine situations such as handling, restraining, or transport are events that can induce states of anxiety, distress, depression, or fear [32,33]. Fear is a negative, subjective, and emotional experience derived from the recognition or anticipation of actual danger [34]. The amygdala is the main component of the so-called fear system [32] and is innately present in many domestic species. Nonetheless, a fearful animal is in a state of chronic stress with its corresponding productive and physiological consequences [35]. In fighting bulls, human contact with animals is limited, sometimes until the bull enters the plaza, to preserve the fearfulness and aggressiveness towards people. The above agrees with Daigle et al. [36], who mentions that temperament and human–animal interaction influence the perception and adaptation to various psychological stressors. It has been reported that the reactions derived from fear preserve the integrity of the animal and improve animal fitness. However, as with any other negative mental state, if fear persists, the animal cannot adapt to its environment, and its welfare is compromised [37].

### 1.2. Stressors of Physical Origin

One example of a stressor of physical origin is fatigue due to transport or other causes of strenuous exercise [38], which results in an increased body temperature, heart and respiration rates, and activation of the hypothalamic–pituitary–adrenal axis [39]. In the physical aspect, there is an increase in creatine kinase (CK) activity in the blood, which is due to tissue damage and poor reperfusion of muscle tissue. When performing physical activity, the active muscle requires oxygen and reserves glycogen energy. However, when the intensity of physical activity increases, the oxygen demand also increases, exceeding the transport system’s levels. In this condition the active muscle use energy from a different source (anaerobic) and the concentration of lactic acid is increased, which, in turn, develops a metabolic acidosis that can lead to the breakdown of the muscle fiber. In addition, CK concentration increases in the blood since it is responsible for maintaining energy homeostasis in sites with high ATP content. Creatine kinase has been used as a biomarker of physical stress and/or muscle damage in animals [40]. Thus, physical stress promotes the inhibition of motor function when the limit of muscular demand is reached. Therefore, CK predominates in physical efforts of high intensity and short duration, such as transportation and vigorous exercise that fighting bulls develop. This could trigger high enzymatic activity. Purroy et al. [41] set out to identify possible muscular pathologies in fighting bulls, and to determine whether they are related to the weakness they show as the bullfight proceeds. In serum samples drawn after the event, they identified increases in the enzymatic activity of creatinine kinase, lactate dehydrogenase, and aspartate transaminase. Moreover, approximately 78% of the bulls sampled in that study presented some histological lesion in skeletal or cardiac muscles with predominant, chronic lesions [41].

### 1.3. Physiological Responses to Stressors

Stress responses consist of a series of physiological and behavioural mechanisms designed to promote adaptation and restore homeostasis in the individual [42], including physiometabolic changes such as tachycardia, hypertension, and hyperthermia [43]; changes that are detectable in animals’ immunological and behavioural responses; electrolyte imbalances; and molecular deficiencies that increase the incidence of oxidative stress, cell death, and DNA alterations [44,45]. As occurs in other mammals, this physiological response to stress begins with activation of the hypothalamic–pituitary–adrenal axis (HPA), which triggers multiple reactions when the central nervous system (CNS) perceives a potential danger. This, in turn, causes alterations of the autonomous nervous system (ANS), and the neuroendocrine disorders described above [46,47,48].

In *Bos indicus*, excitable Brahman heifers had significantly higher serum cortisol concentrations than docile ones, which negatively affected serum LH concentrations [49]. Similarly, Curley et al. [50] found a positive correlation between temperament and cortisol values. The exercise that fighting bulls perform during the 15 min that an average fight lasts [51] and the low aerobic resistance characteristic of bulls could lead their metabolism towards an anaerobic process [52]. In relation to this, Escalera-Valente et al. [51] observed the physiological response in blood samples drawn from 314 4–5-year-old fighting bulls that died after fights characterised by intense exercise. They found that some responses had decreased (blood pH, HCO_3_, BE, PO_2_, sO_2_), others remained within normal ranges (Na^+^, K^+^, iCa, Htc), and the rest increased (PCO_2_, Hb, lactate) compared to normal reference values for other bovine species. Clearly, these events could trigger multiple metabolic responses in fighting bulls, including decreases in the acid–base balance and blood pH [52], as occurs in other animals under similar conditions. However, it is important to point out that, due to the handling procedures used with these animals, the researchers were unable to draw samples before the event that would have permitted a comparative analysis [51]. It is well known that aggressive bovines such as Angus-cross steers can show elevated values of certain metabolites associated with energy catabolism [22], so it is necessary to conduct more studies with fighting bulls to determine whether the values reported by Escalera-Valente et al. [51] could be considered normal due to the temperament of this breed, regardless of the exercise performed during bullfights.

### 1.4. Behavioral Responses to Stressors

Animals modify their behavior as a defense mechanism to cope with or avoid stressors [53]. The behavioral changes can include flight, fight, or freezing, associated with an increase in the concentration of adrenaline or cortisol. Examples of stressful stimuli are a new environment, transportation, vibration, noise, and duration of the trip [54], as well as being exposed to adverse weather conditions [55].

Cattle can perceive sounds of much higher frequencies than humans, and may perceive the noise in the fighting ring as a threat, which is another stressor that can affect their behavior, producing fear [56], especially if when joined with novelty and other negative experiences. Other behaviors associated with fear are increased elimination patterns [57].

The vocalizations of the animals can provide an important source of information about its physical and psychological condition [58]. For this reason, the vocalization structure has been studied as an essential behavioral indicator of their stress level. Low-intensity and lower-pitched vocalizations have been associated with higher cortisol concentrations under stressful events [59].

### 1.5. The Aim of the Review

In this context, the aim of this review is to present current knowledge on the factors that cause stress in fighting bulls during bullfights, including their dying process, by discussing the neurobiology and physiological responses to which they are subjected. Due to the scarcity of scientific studies of these topics, comparisons to other breeds of cattle are included where appropriate.

## 2. Neurobiology of Pain

### 2.1. Pain Perception

**Figure 1:**
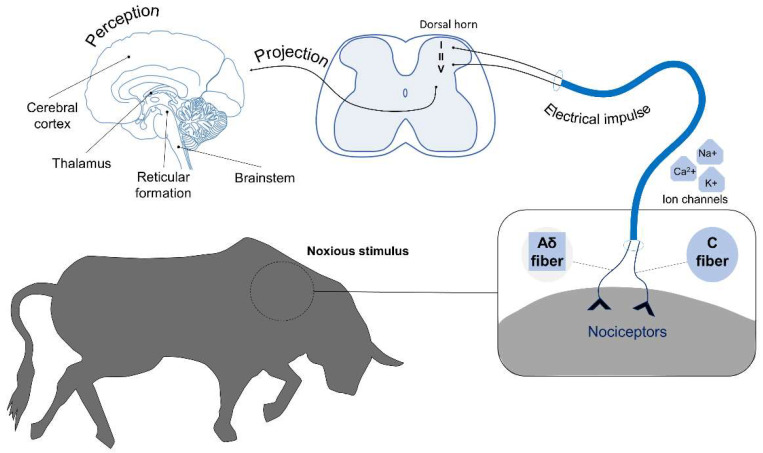
Illustration of the route followed by the nociceptive transmission from the peripheral nerves to the CNS after the initial reception of the harmful stimulus during a bullfight (i.e., placing of the *banderillas*). The process includes transformation of the harmful stimulus into an electrical impulse generated by nociceptors in the skin and muscles that generate the aperture of Ca^2+^, K^+^, or Na^+^ ionic channels to create the electrical impulses that travel through neuronal axons to carry the nociceptive signal to the spinal cord. In this process, information is transmitted through two primary afferent nociceptive neurons called Aδ and C fibres to the dorsal horn of the spinal cord and is then projected by electrical impulses and brainstem to the thalamus, reticular formation, and cerebral cortex, where the pain is perceived.

Animals’ brains are irrigated through the basioccipital plexus and carotid arteries, which supply blood primarily to the occipital lobe of the cerebral cortex, and the basilar arteries that carry blood rostrally [60,61]. During bullfights, bulls are subjected to injuries because the lance (*puya*) and flags (*banderillas*) are stabbed into their bodies, damaging skin, muscles, arteries, veins, and connective tissue, all of which contain physiological sensors called nociceptors. These sensors generate electrical impulses that send signals to the central nervous system, where cattle could detect them as pain [60,62,63]. This sensory information is transmitted from the reticular formation to the thalamus, and from there to the cerebral cortex, where the sensation of pain is finally perceived [19]. The processes involved in pain perception include transduction, transmission, modulation, projection, and perception. Transduction corresponds to the transformation of the harmful stimulus (in this case, mechanical) into an electrical impulse [64] generated by nociceptors in the skin, muscles, bones, or viscera [65]. When activated, these nociceptors generate the aperture of Ca^2+^, K^+^, or Na^+^ ionic channels to create the electrical impulses that travel through neuronal axons to carry the nociceptive signal, successively, to the spinal cord, brainstem, thalamus, and cerebral cortex [66]. In this process, information is transmitted through Aδ nerve terminals that can be nociceptive or nonnociceptive and are composed of low-threshold (<75%) and high-threshold (>25%) mechanoreceptors and mechanothermal receptors. The latter are referred to as Aδ heat nociceptors. High-threshold Aδ nociceptors respond only to tissue-threatening or tissue-damaging stimulation. Many of the Aδ nociceptors respond only to specific stimuli, whereas others are polymodal and respond to mechanical, chemical, and thermal stimulation [67]. In addition, according to Basbaum et al. [68], first and second pain refers to the immediate and delayed pain responses to noxious stimulation. Other terms that denote these pains are fast and slow pain or sharp/pricking and dull/burning pain. The stimuli that generate first pain are transmitted by A-delta, small, and myelinated afferents. Second pain results from the activation of C fibres, which conduct impulses much more slowly, thus accounting for the time difference. Reaction times to first and second pain are about 400–500 and 1000 ms, respectively. Lesions trigger the release of the proinflammatory cytokines (prostaglandins, leukotrienes, bradykinin, serotonin, histamine, substance P) that constitute the so-called “inflammatory soup” [69]. This “soup” can cause, or intensify, nociceptive impulses that facilitate pain transmission [65]. Transmission is followed by modulation, which begins when the stimulus is carried to the dorsal horn of the spinal cord in Rexed laminae I, II, and V [70]. These events stimulate various brain regions, including the cerebral cortex and reticular formation, which transmit sensory information from the thalamus. This is the point at which the perception of pain in the thalamus and cerebral cortex occurs through the spinothalamic and spinoreticular tracts [19,71] (Figure 1).

### 2.2. Emotions and Pain

Some studies of beef and dairy cattle have used the extension of eye white and ear position as indicators when evaluating the emotional states of animals. Battini et al. [72], for example, analyzed 430 photographs of the heads of dairy cows classified in four levels according to the degree of eye opening and ear position. For the latter indicator, drooping ears indicated greater relaxation, while an upright ear position suggested greater excitation. This model was tested under different conditions: during feeding, while at rest, and while grazing, complemented by an avoidance distance trial at the feeding place (ADF). Their findings showed that when the animals were relaxed, their eyes tended to remain half-closed and their ears drooped (67.8% of half-closed eyes, 77.3% with ears drooping or backward, while grazing). In the case of excitation, in contrast, the white surface of the eye increased in extension and was more visible (excitement during the ADF test showed 44.8% of eye white clearly visible), and the ears were pushed forward towards the approaching evaluator (95.5%). Those results support using eye white and ear posture as reliable indicators of emotions in dairy cows. The eye white indicator was also tested by Core et al. [73] to predict temperament in a herd of cattle. The 147 animals studied were a mix of British (predominantly Angus), Continental (predominantly Simmental), and Piedmontese breeds. They were grouped as heifers (n = 48), bulls (n = 39), and steers (n = 60), and then videotaped while in a squeeze chute where they were selected. Chute temperament scores were assigned as follows: 1 (calm) to 5 (agitated), and the eye white area was expressed as the percentage of exposed eye area. Those authors found the highest average percentage of eye white in the bulls (31.43 ± 14.77), followed by the heifers and steers (30.14 ± 14.37 and 28.57 ± 12.38, respectively). The Pearson correlation coefficients for eye white percentage and chute temperament scores were 0.95 for bulls (*p* < 0.0001), 0.674 for heifers (*p* < 0.0001), and 0.696 for steers (*p* < 0.0001). Thus, they concluded that the percentage of eye white in cattle can be used as a quantitative tool that requires minimal equipment to assess temperament in beef cattle, and that it provides an objective method for temperament selection. These indicators might, therefore, also be used as non-invasive tools for evaluating the degree of excitation in fighting bulls, though under different conditions.

Fighting bulls are reared in extensive environments with minimal exposure to humans. Fear is arguably the most investigated emotion in domestic animals. It is a potent stressor. The highly variable results are likely due to different levels of physiological stress such as fear stress, including handling, contact with people, or exposure to novelty. However, we lack scientific studies of this breed that evaluate the degree of expression of positive and negative emotions under different conditions. Since the stimuli that can cause fear in bulls—and other animals—during fights include confronting a closed, unfamiliar environment, isolation, separation from conspecifics, exposure to predators or aggressors, the absence of escape routes or refuge, and the presence of harmful stimuli in conditions that preclude escape [74]. Pain and emotion are part of a more extensive motivational system that promotes survival, and the neurocircuitries associated with emotion and pain overlap significantly [75].

### 2.3. Analgesic Effects

It is possible, however, that the stress provoked could inhibit the transmission of pain stimuli in the brain and spinal cord [76]. To become effective, this pain reduction process must be activated by the amygdala. This involves endogenous opioids that modulate signalling and synaptic transmission in the neural loci that contribute to the experience of pain [77]. The genetic makeup and aggressive behaviour typical of fighting bulls during events leads them to adopt a challenging attitude as they confront their attacker, making no attempt to flee from the situation. The activation of neuroendocrine mechanisms allows release of the hormone proopiomelanocortin (POMC), β-endorphins, and methane cephalins, cortisol, and ACTH in response to stress. Centenera [78] took blood samples from fighting bulls at four stages of the event: immediately upon entering the ring (n = 159 bulls), after the wounds inflicted by the lance (n = 137 bulls), after the placing of the *banderillas* (n = 110 bulls), and at the end of the fight when the bull is killed (*estoque*) (n = 80 bulls). Their post-event findings showed an increase in the concentration of POMC—a precursor hormone of the β-endorphins and methane cephalins—that was six times higher in the animals after the *estoque* compared to the concentrations determined when the bulls entered the ring (*p* < 0.01). With respect to serum ACTH and cortisol levels, that study found higher concentrations in the bulls immediately after leaving the ring, while the lowest values were determined for the samples drawn and analyzed after the final *estocada* (four and three times lower, respectively) (*p* < 0.01).

## 3. Muscle-Skeletal Injuries during Bullfights

According to Fernández and Villalón’s [11] anatomical review, fighting bulls lack clavicles, so their two anterior extremities are joined at the trunk, mainly by muscles. The scapula has a prolongation cartilage where those muscles are inserted to join the two extremities more strongly and fix them to the trunk. Muscle fibres, of course, are classified histologically in various types according to the relation between myosin adenosine triphosphatase activity (m-ATPase) and pH [79]. When pH is alkaline, type I muscle fibres (slow-contracting) have low m-ATPase activity, while type II fibres (fast-contracting) have high m-ATPase activity [80]. Under conditions of intense exercise, such as a bullfight, the fast-contracting muscle fibres with low oxidative capacity (type II) are the main ones that function to produce anaerobic glycolysis as a pathway for producing the energy required for the effort involved. During this process, either pyruvate is formed and used by the mitochondria, or lactate is produced, which is (partly) delivered to the blood stream. From there, it reaches the liver and kidneys that convert it to glycogen, as occurs in other mammal species, including humans [81,82,83,84]. The enzyme lactate dehydrogenase catalyses the interconversion of pyruvate and lactate. However, when lactate is abundant, it remains detectable and indicates recently performed heavy physical activity. Increased physical activity may also induce damage to muscle fibres and the release of creatine phosphokinase (an enzyme used by the muscular tissue for produce creatine) into the blood [85]. During bullfights, bulls are subjected to anatomical injuries such as torn muscles, ligaments, tendons, and ruptured nerves and blood vessels caused by the bullfighters’ weapons [11]. Other injuries that may occur include fractures of the ribs, the spinous processes on the vertebrae, and prolongation cartilages [11] that could cause severe pain and changes in the animal’s neurobiology.

Gomariz et al. [12] attempted to determine the causes of the physiological disequilibrium of the locomotor apparatus by evaluating various transversal cuts of several muscles—common digital extensor, long digital extensor, long thorax, Latissimus dorsi, Ventral thoracic serrate, and gluteobiceps—from six fighting bulls killed by the matador’s sword that presented an obvious lack of strength before death, manifested in frequent falls recorded in their movement profile, as Table 1 shows. They used histological and histochemical techniques, stained their samples and then microphotographed them at 10×, 20×, and 40×. Findings allowed them to identify the following lesions: mitochondrial alterations, loss of the polygonal contour of fibres, centralization of nuclei, necrotic processes, fibrillar fragmentation, and vacuolization of the sarcoplasm. In some subjects, the injuries examined were accompanied by alterations of the connective tissue (peri and endomysial fibrosis). The authors concluded that this series of injuries could be a consequence of the excessive muscular effort that the bulls made in a short time-period. They did not rule out the possibility that some of the animals may have suffer from a myopathy. Whatever the case, they suggest that the lesions affected muscle fibres and connective tissue, leading to a loss of strength and frequent falls during the bullfights.

In addition to the injuries visible at first sight during a bullfight, there are conditions in fighting bulls that could exacerbate muscular and skeletal damage. The study of 120 fighting bulls by Lomillos-Pérez and Alonso de la Varga [86] detected the presence of osteochondrosis in over 70% of the animals evaluated, bilaterally in 78.3% of them. Various authors identify osteochondrosis as an element that predisposes fighting bulls to develop the so-called “falling syndrome” [87,88], an affliction characterized by loss of equilibrium and transitory falling that has also been associated with damage to muscle cells [12].

Martínez [89] and Lomillos-Pérez et al. [90] reported that causes of the falling syndrome can include genetic factors, transport conditions, the physical demands of the bullfight, a lack of functional exercise, alimentary deficiencies, and circulatory, nervous, metabolic, endocrine, or etiological disorders. According to Lomillos-Pérez et al. [90], this syndrome has decreased over time, as incidence has decreased from 99.56 to 79.82%, and that it is in the third (cape) stage of the bullfight that it occurs most frequently. Even though the incidence remains very high, it is noteworthy that this partial decrease in incidence occurs during the *banderillas* stage. Dávila et al. [91] point out that any discussion of the aetiology of osteochondrosis in fighting bulls must mention the trauma and biochemical elements of the cartilage, which can be affected by nutritional deficiencies, hormonal imbalances, inadequate vascular contribution, and genetic factors.

## 4. Hypovolemic Shock

The wound inflicted by the lance causes a loss of blood volume, the first event in a series that ends in hypovolemic shock [10]. Hypovolemia is the reduction of blood volume due to massive haemorrhaging that induces severe dehydration. In this condition, both the amount of blood that reaches the body’s vital organs and the pressure with which it arrives are insufficient, impeding their functioning and viability [92]. Three phases of hypovolemic shock have been described: compensatory, in which the organism generates a neuroendocrine response as it struggles to maintain haemodynamic status; decompensatory, when it sustains continuous hypoperfusion that triggers a process of cell injury and death; and microcirculatory dysfunction, when the parenchymal tissue is damaged and inflammatory cells are activated [93]. This condition is partially compensated at onset by the release of K^+^ ions from the intracellular space to the blood. This mechanism aims to self-compensate and cause isotonic dehydration and hyperkalaemia, but the resulting hydroelectrolytic imbalance produces vascular dysfunction. At the same time, other compensating mechanisms are activated to lower arterial pressure. This is detected initially by baroreceptors in the aortic arch and carotid sinus, leading to activation of the sympathetic system that secretes catecholamines, angiotensin II, and the antidiuretic hormone to preserve cardiac output and maintain adequate cerebral and cardiac perfusion [94].

Other essential responses of the fighting bull’s organism during a bullfight include splenic contraction, when erythrocytes are mobilized towards the zones where additional oxygen support is required with increased haematocrit due to the dehydration the animals may present as a consequence of the intense physical activity performed in a short period [51].

## 5. Metabolic Responses Linked to Psychological Stress and Physical Exercise

Animals are subject to various environmental and behavioural stressors that affect their survival and physical state [95]. To respond physiologically to these stressors, they present a series of neural and endocrine responses that divert energy away from short term, non-essential physiological processes such as growth, digestion, and reproduction, in an effort to resolve the stressful situation. Meanwhile, the neural stress response involves secreting catecholamines from the adrenal medulla and the sympathetic nervous system, and mobilising energy to increase cardiac frequency, blood pressure, and respiration [96,97].

The exertion demanded of bulls during the 15–18 min that bullfights usually last can be considered similar to that performed by athletic animals forced to perform enormously intensive exercise [52,98]. This explains why acid–base balance alterations are observed that lower blood pH [51]. Under these conditions, blood pH can decrease to levels below 7.2, aerobic glycolysis is inhibited, extracellular osmolarity increases, and cellular oedema may occur. It is well known that increased acidity can produce a broad range of harmful effects on neural functioning, such as increasing the permeability of the blood–brain barrier, inhibiting mitochondrial function, and altering synaptic transmission and ionic functions [99]. Among the mechanisms that the organism has at its disposal to eliminate hydrogen ions and maintain pH, we can mention several buffering systems, such as the respiratory and buffer bases [100].

**Figure 2:**
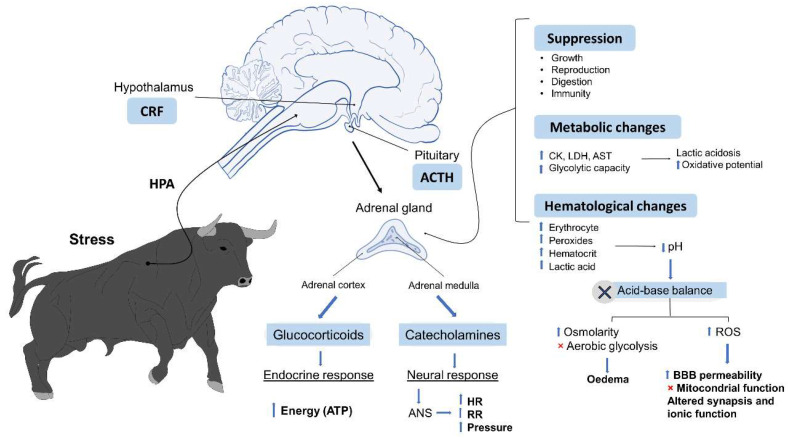
Metabolic, haematological, and acid–base balance alterations that occur during the endocrine response to stress and the physical effort in fighting bulls. One of the main metabolic responses that occurs during stressful conditions consists in an increase of adrenal glucocorticoids, such as cortisol, that circulate in the bloodstream [95]. In other species, it is well known that short periods of glucocorticoid release can cause irreversible damage, including reproductive disorders, immunosuppression, and reduced life expectancy [96,114,115]. In addition, the emotional stress and intense exercise that fighting bulls undergo and the exposure to a new environment during the event produce marked increases of cortisol, glucose, and T3 in the bloodstream that can generate significant biochemical changes in the organism by triggering the stress-adaptation syndrome [52]. Catecholamines function to prepare an organism for the “flight-or-fight” response but triggers tachycardia, hypertension, hyperthermia, hyperventilation, and sweating [48,116]. Cortisol begins to be secreted by the adrenal cortex around five minutes after the stressful stimulus is presented. This substance, which can be detected in blood, saliva, urine, and faeces, performs the primary function of increasing and then maintaining blood glucose levels using reserves of hepatic and muscular glycogen to provide the animal with sufficient energy to sustain the physical effort that the situation demands [117]. CRF: corticotropin releasing factor; HPA: hypothalamic-pituitary-adrenocortical axis; ACTH: adrenocorticotropic hormone; ATP: adenosine triphosphate; ANS: autonomic nervous system; HR: heart rate; RR: respiratory rate; CK: creatine kinase; LDH: lactate dehydrogenase; AST: aspartate aminotransferase; ROS: reactive oxygen species; BBB: blood–brain barrier.

Bullfights demand an enormous physical effort by the bulls, so these animals must be in optimal health conditions before participating. The intensity of the fight triggers significant metabolic alterations that are observable after the event, including haematological changes (increased red blood cells and haematocrit), and elevated peroxides and lactic acid production that reduce concentrations of muscle glycogen and pH [101]. Accordingly, Lacourt and Tarrant [102] and Agüera et al. [103] showed that the physical and emotional stress and exercise to which fighting bulls are subjected during an event causes a marked reduction of glycogen in type I and type II fibres. These changes are accompanied by the release of large amounts of enzymes into the bloodstream, including creatinine kinase (CK), lactate dehydrogenase (LDH), and aspartate aminotransferase (AST) [52]. The antemortem analysis of certain biological variables in animals can be useful for diagnosing diseases or detecting metabolic states [104]. Fighting bulls are known for their aggressiveness and natural resistance to handling, so drawing in vivo blood samples can be extremely difficult [105]. Although the emotional and physical stress that these bulls experience during bullfights can cause significant changes in blood analyte levels (Figure 2), blood variables are influenced by physical exertion and stressful situations; consequently, post-mortem blood analysis does not reflect basal concentrations for this species. Therefore, these indicators cannot always be used as diagnostic findings in post-mortem blood evaluations [106]. However, ocular fluids such as vitreous humour maintain a stable composition after death and can be used post-mortem to estimate the blood levels that animals presented antemortem. González-Montaña et al. [105] used post-mortem ocular fluids in fighting bulls, finding that all the variables assessed in plasma showed concentrations above basal levels. Specifically, alterations were observed for glucose, uric acid, LDH, and creatinine kinase (CK). These findings can be caused by the overexertion, stressful situation, destruction of muscle cells, and loss of bodily fluids that the bulls undergo during the intense exercise of a bullfight. Several studies of pigs and horses showed that animals performing high levels of physical activity and training have a corresponding higher oxidative capacity, higher glycogen content, and larger amounts of type II muscle fibres than animals that perform less physical activity [106,107,108,109]. It seems that the metabolic capacity of bulls varies according to age. A study of young and mature fighting bulls by Agüera et al. [103] analysed the values of citrate synthase (CS), 3-hydroxyacyl coenzyme A dehydrogenase (HAD), LDH, glycogen, lactate, and pH in biopsies of the gluteus medius muscle obtained after bullfights. They observed that HAD and LDH activity were higher in the group of older bulls. Glycogen concentrations and pH were low in both groups, but lactate concentrations were higher in the older bulls. These results show that young and old bulls have similar muscle fibre type composition but the metabolic capacity differs, with a higher glycolytic capacity and lactate production in older bulls [103]. In another study, Purroy and Buitrago [110] observed that the levels of CK, oxalacetate glutamate transaminase (GOT), and LDH were higher post-mortem because the animals had been subjected to more intense exercise in the days leading up to bullfights. When the data obtained after exercise in fighting bulls were compared to normal reference values for cattle, it was clear that some blood variables—pH, bicarbonate (HCO_3_^−^), base excess (BE), oxygen partial pressure (PO_2_), and oxygen saturation (sO_2_)—decreased, while others—sodium (Na^+^), potassium (K^+^), calcium ion (iCa), and haematocrit (Htc)—remained within normal limits [51]. Other analytes, such as PCO_2_, haemoglobin (Hb), and lactate, were above normal values. A study by Muñoz-Juzado et al. [111] evaluated the oxidative and glycolytic potential in muscle biopsies of fighting bulls taken after an event. Samples were drawn from the *gluteus medius* and *semitendinosus* muscles of bulls aged 1 to 3 years. Those authors found that the highest oxidative muscular potential was manifested in the 2-year-old bulls and that glycolytic capacity increased progressively with age. This contrasts with other bovines, where a reduction in the oxidative potential occurs from the time of birth onwards [102]. These findings lead to the suggestion that the age of the bull might participate significantly in the metabolic responses during bullfights, as it does in muscle enzyme production. Physiological responses are the reactions to stressful stimuli that occur in organisms. Heart rate is the most useful parameter for evaluating the activation of the flight-or-fight syndrome [103]. When correlated with body temperature, it can be interpreted as the heart’s response to metabolic demand [92]. Likewise, skin temperature is a useful parameter for evaluating vascular resistance, vasodilatation, and vasoconstriction. When body temperature decreases distally, vasoconstriction is present with low cardiac output and likely, hypovolemia [112]. If, in contrast, the temperature tends to increase towards distal areas, vasodilatation is occurring with high cardiac output [113]. Finally, to compensate the condition of metabolic acidosis, animals present hyperventilation or tachypnea, which can be detected by the flaring or flapping of their nostrils and more evident inspiratory movements of the abdominal and thoracic walls.

According to García-Belenguer et al. [118], fighting bulls present low selenium and vitamin E levels but high copper levels in the blood, possibly associated with exercise during the fight. Carpintero et al. [119] identified that calcium, phosphorus, and magnesium levels are well above normal physiological values after bullfights. They attributed hypercalcaemia and hypermagnesaemia to dehydration during fights and the finding of hyperphosphatemia to respiratory and lactic acidosis. After a bullfight, high magnesium and phosphorus levels were reported by González-Montaña et al. [120] in 15 fighting bulls aged 4–5 years, based on measurements of the vitreous humour, aqueous humour, and blood. They also determined that these levels were higher in blood plasma than in the vitreous humour, while calcium, chrome, and sodium levels were similar in all three fluids. Selenium, iron, zinc, and copper values were 16–32 times higher in plasma than in the ocular fluids. In summary, studies have found that changes at the muscular level and in diverse body fluids result from the physiological effort and energy demand to which fighting bulls are subjected during bullfighting events [103,111]. The most significant changes from the perspective of animal welfare include those related with psychological stress [121] with negative emotions, including fear, pain, and triggering physiological responses, including dehydration, hypermagnesaemia, hypotension, muscular necrosis, myoglobinuria, and metabolic acidosis.

## 6. Conclusions

The existing information allows us to conclude that bulls face energy and metabolic demands during bullfights due to the high intensity and duration of the exercise performed, together with muscular injuries, physiological changes, and high enzyme concentrations. In addition, the final stage of the bullfight causes a slow dying process for an animal that is sentient and conscious of its surroundings. Unfortunately, due to the scant literature on this breed, many gaps exist in the available information; more specific information, such as physiological evaluations, could help verify these effects.

## Figures and Tables

**Table 1:** Contribution of muscles to movement in fighting bulls.

Muscle Group	Function
Common digital extensor, gluteobiceps, and long digital extensor	Support in extending and retracting extremities
Long thorax	Fixing and righting action of the rachis; dorsal flexor agent of the thoracic-lumbar rachis; regulating mechanical influences in the protraction–retraction of pelvic members
Latissimus dorsi	When contracted, once the protraction of the thoracic member is culminated (support in extension); drags body mass while retraction of the member lasts
Ventral thoracic serrate	Constitutes the principal suspensor agent of the trunk.
From Gomariz et al. [12]	

## Data Availability

Not applicable.
